# Exploiting tumor epigenetics to improve oncolytic virotherapy

**DOI:** 10.3389/fgene.2013.00184

**Published:** 2013-09-20

**Authors:** Nicole E. Forbes, Hesham Abdelbary, Mathieu Lupien, John C. Bell, Jean-Simon Diallo

**Affiliations:** ^1^Center for Innovative Cancer Research, Ottawa Hospital Research InstituteOttawa, ON, Canada; ^2^Faculty of Medicine, University of OttawaOttawa, ON, Canada; ^3^Ontario Cancer Institute, Princess Margaret Cancer Center/University Health NetworkToronto, ON, Canada; ^4^Ontario Institute for Cancer ResearchToronto, ON, Canada; ^5^Department of Medical Biophysics, University of TorontoToronto, ON, Canada

**Keywords:** oncolytic virotherapy, epigenetic modulation, cancer, tumor heterogeneity, anti-viral response, antigen presentation

## Abstract

Oncolytic viruses (OVs) comprise a versatile and multi-mechanistic therapeutic platform in the growing arsenal of anticancer biologics. These replicating therapeutics find favorable conditions in the tumor niche, characterized among others by increased metabolism, reduced anti-tumor/antiviral immunity, and disorganized vasculature. Through a self-amplification that is dependent on multiple cancer-specific defects, these agents exhibit remarkable tumor selectivity. With several OVs completing or entering Phase III clinical evaluation, their therapeutic potential as well as the challenges ahead are increasingly clear. One key hurdle is tumor heterogeneity, which results in variations in the ability of tumors to support productive infection by OVs and to induce adaptive anti-tumor immunity. To this end, mounting evidence suggests tumor epigenetics may play a key role. This review will focus on the epigenetic landscape of tumors and how it relates to OV infection. Therapeutic strategies aiming to exploit the epigenetic identity of tumors in order to improve OV therapy are also discussed.

## INTRODUCTION

While genetic information establishes the primary blueprint for cellular identity, multiple regulatory layers responsive to extra and intra-cellular signals ultimately control the manifestation of this blueprint. Changes in cellular state, including initiation of DNA synthesis, activation of apoptotic programs, or triggering of antiviral defense mechanisms, result from an integrated response to stimuli received by the cell. These are controlled in large part by gene/protein expression profiles unique to each cell. It is now well understood that activation of transcription factors that bind in a DNA sequence-specific manner at promoter and enhancer elements is responsible for many of the changes in gene expression that occur in response to environmental or developmental cues. However transcription factors and their associated gene targets are themselves further regulated by the accessibility of DNA sequences. Since the genome resides in the finite space provided by the nucleus, it interacts with proteins known as histones to form chromatin and facilitate its compaction. The configuration of chromatin compaction is modulated by epigenetic modification and is a key determinant for transcription factor-mediated activation of gene transcription (Magnani et al., 2011).

Epigenetic modifications create a reversible imprint that may be inherited through cell division. For example, DNA methylated at promoter CpG islands is associated with gene silencing and can be reversed by treatment with DNA methyltransferase inhibitors such as 5-AZA (5-aza-2′-deoxycytidine) leading to the reactivation of silenced genes (Baylin and Jones, 2011; Krecmerova and Otmar, 2012). Similarly, chromatin structure can alter accessibility to the DNA template and can be readily remodeled by histone post-translational modifications (PTMs). PTMs including acetylation, methylation, phosphorylation, ubiquitination, and many others can be added to numerous residues of histone proteins (Bannister and Kouzarides, 2011). Different PTMs will favor chromatin compaction while others will increase its accessibility to DNA binding proteins. Histone modifications and DNA methylation are highly interdependent processes and define the epigenetic code (Cedar and Bergman, 2009). The epigenetic code is regulated by a complex interplay of enzymatic erasers, readers, and writers that exhibit specificities toward different histones and residues (Rice and Allis, 2001). For example, the level of histone acetylation is regulated by the relative activity of histone acetyltransferases (HATs) and histone deacetylases (HDACs), proteins with opposing enzymatic activities that are often found in the same protein complexes (Johnsson et al., 2009; Peserico and Simone, 2010). This also applies to histone lysine methyltransferases (KMTs) and lysine demethylases (KDMs). Consequently, modulating the activity of histone-modifying enzymes can profoundly alter the epigenetic profile of a cell (Egger et al., 2004; Yoo and Jones, 2006).

Given their critical role in the regulation of normal cellular physiology, it is not surprising that aberrations in epigenetic modifications can contribute to the manifestations of human disease. For example, a cell’s epigenetic profile can impact the progression of acute microbial diseases (discussed in more detail below) as well as the development and treatment of chronic diseases such as cancer. DNA hypermethylation is often observed in cancer cells (Patel et al., 2012). The genome-wide distribution of histone modifications can also be altered in the course of cancer development (Akhtar-Zaidi et al., 2012; Magnani et al., 2013). As well, the activity of various histone-modifying enzymes can be altered through mutations (Taylor et al., 2011), aberrant expression (Schildhaus et al., 2011; Bennani-Baiti et al., 2012) and/or recruitment to target histone residues via oncogenic fusion proteins (Lubieniecka et al., 2008). Consequently, many cancers are sensitive to epigenetic modulators such as 5-AZA, HDAC, or KDM inhibitors (Hurtubise et al., 2008; Taylor et al., 2011; Schenk et al., 2012) and epigenetic modifications have been shown to influence the response to chemotherapy (Glasspool et al., 2006; Magnani et al., 2013).

## ONCOLYTIC VIROTHERAPY

While epigenetic modulators hold promise as anticancer agents, it is clear that like for many other cancer therapies, tumor-specificity is of paramount importance. Tremendous efforts have been made over the past decades to tackle the difficult task of developing more selective cancer therapies, aiming to exploit the sometimes-subtle differences between normal tissues and tumors. One promising new class of therapeutics comes to us from the field of virology. Since the early 1900s it has been observed that cancers can be uniquely susceptible to virus infection (Dock, 1904). While the first clinical trials using replication-competent viruses to treat cancer began in the seventies (Asada, 1974; Kelly and Russell, 2007; Pol et al., 2013), approval of the first oncolytic virus (OV) is only now in the foreseeable future in North America (Carroll, 2011; Galanis et al., 2012; Heo et al., 2013). The more recent clinical success of OVs is in large part due to our more complete understanding of the molecular biology of both cancer cells and viruses that allowed us to create virus strains with improved selectivity and anti-tumor activity, and clinical safety profile (Breitbach et al., 2011). Rapid proliferation and deregulated metabolism (Fritz and Fajas, 2010), disorganized vasculature (Jain, 2005), and defective antiviral innate immune responses (Dunn et al., 2006) in malignant tumors are hallmarks that not only define cancer, but also favor viral growth. Building on these observations, several OVs have been engineered or selected to take advantage of one or more of these features (Russell et al., 2012). A variety of OV platforms are currently under clinical evaluation including those based on herpes simplex virus (HSV), Reovirus, vaccinia virus (VV), Adenovirus, Measles virus, and vesicular stomatitis virus (VSV; U.S. National Library of Medicine, 2013).

## ONCOLYTIC VIROTHERAPY AND THE CELLULAR INNATE ANTIVIRAL RESPONSE

It is now well established that cancer cells that evolve to frank malignancies often acquire defects in their ability to mount a successful antiviral response and this attribute/deficit contributes to the selectivity of many if not all OVs (Norman and Lee, 2000; Stojdl et al., 2000, 2003). This is often a consequence of the observation that approximately 65–70% of tumors are unable to produce or respond to type I interferon (IFN), a key mediator of the cellular antiviral response (Stojdl et al., 2003; Dunn et al., 2006). IFNs are antiviral cytokines induced following recognition of viral proteins and nucleic acids by cellular pattern recognition receptors such as Toll-like receptors (TLRs) that signal through to transcription factors such as interferon regulatory factors (IRFs). There are many isoforms of IFN, which can be functionally sub-divided in at least three types (types I/II/III). While type I/III IFNs (e.g., IFN-α, IFN-β/IFN-λ) stimulate cellular antimicrobial immunity; type II IFNs (e.g., IFN-γ) coordinate the host immune response. IFNs elicit their transcriptional effects through autocrine and paracrine activation of IFN receptors and signaling through the Jak/STAT signaling pathway (Borden et al., 2007). This induces the transcriptional up-regulation of interferon-stimulated genes (ISGs), many of which have direct antiviral/pro-apoptotic activities (e.g., RNAseL, TNF-α, TRAIL) and/or immune-stimulatory properties (e.g., components of major histocompatibility complex).

## ONCOLYTIC VIRUSES AND THE GENERATION OF AN ANTI-TUMOR IMMUNE RESPONSE

In addition to taking advantage of a niche provided by aberrations unique to cancer and the tumor microenvironment, OVs have been used as platforms to express a range of therapeutic transgenes, from suicide genes to immune-stimulatory cytokines (Merrick et al., 2009; Maldonado et al., 2010; Chai et al., 2012; Stephenson et al., 2012; Lange et al., 2013). In this regard, it is now well recognized that beyond simply lysing infected tumor cells, OVs effectively “de-cloak” tumors by stimulating immune cells to recognize cancer antigens, ultimately leading to tumor destruction and in some cases, long-term cures (Sobol et al., 2011; Huang et al., 2012). Many tumors evade immune recognition due to a dysfunctional antigen presentation pathway, which is under tight multilayered transcriptional control ultimately dictated by type I/II IFNs and the class II transactivator (CIITA). This transcription factor controls the expression of numerous genes involved in antigen presentation, including class I and II MHC molecules, which display tumor or pathogen derived peptides to killer T cells (CD4^+^/CD8^+^; LeibundGut-Landmann et al., 2004).

The antigen presentation pathway is influenced by both tumorigenesis and OV therapy. Many tumor cells including leukemias, lymphomas, and carcinomas, avoid immune recognition due to a dysfunctional antigen presentation pathway, largely caused by epigenetic silencing (e.g., histone deacetylation or DNA methylation) of *MHC2TA*, the gene encoding CIITA (LeibundGut-Landmann et al., 2004)*.* OV therapies can enhance tumor-associated antigen presentation through various mechanisms. In response to OV infection, type I and II IFN secretion by infected cells within the tumor environment (which also includes normal tumor infiltrating cells) leads to the up-regulation of hundreds of ISGs including IRF-1, which up-regulates CIITA expression (Muhlethaler-Mottet et al., 1998). Notably, this response is dependent upon the ability to respond to IFN, which can be limited in many cancer cells (Stojdl et al., 2003; Dunn et al., 2006).

Oncolytic virotherapy can have a positive influence on antigen presentation and the anti-tumor response. Some OVs including HSV, reovirus, and measles virus, induce syncytia formation in infected and neighboring cells. These large multinucleated tumor cells secrete an abundance of “syncytiosomes,” which are exosome-like vesicles that present tumor-associated antigens via MHC molecules (Bateman et al., 2000, 2002). Finally, destruction of cancer cells following infection by OVs provides an additional source of tumor antigens available for capture by antigen-presenting immune cells. The immunostimulatory nature of the virus itself, through activation of TLRs and subsequent cellular production of pro-inflammatory cytokines stimulates the recruitment of antigen-presenting cells that sample tumor-derived and virus-expressed antigens. Presentation of tumor antigens to killer T cells (CD4^+^/CD8^+^) through MHC molecules in the presence of inflammatory cytokines can thus lead to generation of a robust and long-lasting immune responses directed against the tumor.

To capitalize on these beneficial immunological effects, some groups have developed OV/vaccine hybrid strategies. These strategies are designed specifically to re-educate the adaptive immune system to recognize and respond to tumor antigens. Thus, OVs can be engineered to express not only immune-stimulatory cytokines but also tumor-specific antigens to further stimulate an anti-tumor immune response following OV infection of cancer cells (Diaz et al., 2007; Pulido et al., 2012). Indeed, several studies have shown that this “tumor antigen vaccination” effect can be further amplified using a prime-boost strategy, by priming with an antigen then boosting the response using an OV expressing the same antigen (Bridle et al., 2010, 2013). As discussed below, it is possible to use epigenetic modifiers to further fine-tune this oncolytic vaccine approach. It is also possible to take advantage of this vaccine effect by infecting cancer cells *ex vivo* and re-injecting the inactivated “oncolysate” to generate prophylactic and even therapeutic anticancer immune responses. The resulting up-regulation of MHCs and co-regulatory factors and presentation of tumor antigens at the surface of OV infected cells as well as the presence of immune-stimulating virus is thought to be at the root of this effect (Lemay et al., 2012). Overall, these studies emphasize the important role of antigen expression/presentation in OV-stimulated anti-tumoral responses.

## TUMOR HETEROGENEITY: INHERENT BARRIER TO OV THERAPY

Despite promising clinical data, it is clear that there is considerable inter- (and likely intra-) tumor heterogeneity in the responsiveness to OV therapy *in vitro* as well as *in vivo* in both pre-clinical and clinical settings (Breitbach et al., 2011; Sobol et al., 2011). Because overcoming the innate cellular antiviral response and generating a robust anti-tumor response are critical to observe meaningful therapeutic benefits from oncolytic virotherapy, it is important to understand what tumorigenic processes influence these closely linked pathways in order to manipulate them to improve therapeutic outcomes.

**FIGURE 1:**
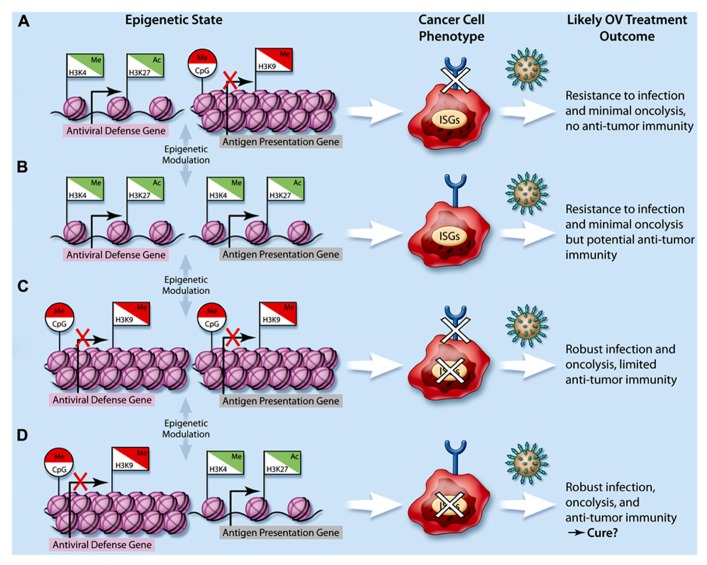
**Impact of cancer epigenetics on oncolytic virotherapy.** The integration of repressive epigenetic marks such as DNA CpG methylation (Me, circle flags) and histone H3K9 methylation (Me, square flags), and activating epigenetic marks such as histone H3K4 methylation and histone H3K27 acetylation (Ac, square flags) lead to higher-order nucleosome packaging and repression (red flags) or open chromatin and gene expression (green flags). In cancer cells, dysregulation of epigenetic processes leads to various possible epigenetic states with respect to genes involved in the antiviral response (e.g., type I IFN, interferon stimulated genes or ISGs) as well as those involved in antigen presentation (e.g., MHC I/II expression, represented by a semi-circle at the end of a stick). This ultimately leads to a variety of cancer cell phenotypes **(A–D)** and subsequently, a variety of potential therapeutic responses to oncolytic viruses (OVs, represented by spiked green circles).

Given the profound epigenetic divergence that prevails in tumor cells (Akhtar-Zaidi et al., 2012; De Carvalho et al., 2012), it is foreseeable that tumor-specific gene expression response profiles induced by virus infection may be altered by epigenetic modifications and that this could contribute to the heterogeneity of tumor responsiveness to OVs. As discussed previously, epigenetic reprogramming is well known to play an important role in oncogenic transformation and numerous reviews extensively cover the role of epigenetics in cancer (Muntean and Hess, 2009; Baylin and Jones, 2011; Hatziapostolou and Iliopoulos, 2011; Suva et al., 2013). Thus, the remainder of this review aims to highlight current knowledge of genes epigenetically regulated in cancer that are also involved in pathways critical for OV therapy, namely the IFN-mediated antiviral response and antigen presentation (**Table 1**), and how this contributes to tumor heterogeneity (**Figure 1**).

**Table 1:** Epigenetic control: implications in cancer and OV therapy.

Genetic target	Cellular function	Epigenetic modification	Cell type	Reference
ISGs (*IFI27*, *9–27*, *LMP2*, *LMP7*, *Viperin*, *IFI44*, *IFIT2*, *ISG56*)	Antiviral response	DNA hypermethylation	Huh-7 cells (Human hepatoma)	Naka et al. (2006)
*STAT1*, *ISGs* (*IFI27,* *IRG1*, *Viperin, Cxcl10, ISG15, IFI44)*	Antiviral response, anti-tumor response, antigen presentation	Histone deacetylation	Human cortical neurons	Cho et al. (2013)
*CREB3LI*, *MX1*	Antiviral response	DNA hypermethylation	Human hepatoma Huh-7 cells	Chen et al. (2013)
*IFN-β*, ISGs (*MX1*, *IFIT1*, among many)	Antiviral response	H3K9 dimethylation	Mouse embryonic fibroblasts, mouse splenic dendritic cells	Fang et al. (2012)
*IFN-γ*	Antiviral response, anti-tumor response	H3K4 trimethylation	Mouse CD4^+^/CD8^+^ T cells	Gomez et al. (2013)
*IRF7*, IFN regulated genes	IFN-β induction, antiviral response	DNA hypermethylation	Li-Fraumeni immortalized cells	Fridman et al. (2006)
*IRF7, IFITM1, OAS1, OAS2, STAT1, MX1, TIP30, IL-8, TRAIL, HLA-F, HLA class I locus C heavy chain,* among others	IFN-α/β induction, antiviral response, anti-tumor response, antigen presentation	DNA hypermethylation	Li-Fraumeni immortalized cells	Kulaeva et al. (2003)
*IRF7*	IFN-α/β induction	DNA hypermethylation	Li-Fraumeni immortalized cells	Li et al. (2008)
*IRF8*	IFN signaling, differentiation, apoptosis, tumor suppression	DNA hypermethylation	Nasopharyngeal, esophageal, breast, and cervical primary carcinomas	Lee et al. (2008)
*IRF4, IRF5, IRF8*	IFN signaling, differentiation, apoptosis signaling, tumor suppression	DNA hypermethylation	Gastric carcinoma	Yamashita et al. (2010)
*STAT1, STAT2,* and *STAT3*	Antiviral response, antigen presentation, anti-tumor response	DNA hypermethylation	Colon carcinoma	Karpf et al. (1999)
*JAK1 kinase*	Antiviral response, antigen presentation, anti-tumor response	DNA hypermethylation, histone deacetylation	Prostate adenocarcinoma	Dunn et al. (2005)
*Apo2L/TRAIL receptor 1 (DR4), RASSFIA, XAF1, TRAIL*	TRAIL-mediated apoptosis	DNA hypermethylation	Melanoma cell lines, renal carcinoma, experimentally transformed human cell lines	Reu et al. (2006a,b), Baeet al. (2008), Lund et al. (2011)
unknown	TRAIL-mediated apoptosis	Histone deacetylation	Medulloblastoma	Hacker et al. (2009)
*IFITM1*	Antiviral response	DNA hypermethylation	Gastric carcinoma	Lee et al. (2012)
ISGs (Global regulation)	Antiviral response, Anti-tumor response	Histone deacetylation	U2OS (osteosarcoma), HeLa (cervical carcinoma)	Chang et al. (2004)
ISGs under ISRE control	Antiviral response, Anti-tumor response	Histone deacetylation	Human foreskin fibroblasts	Sakamoto et al. (2004)
*IFN-β, FGF2, VEGFC, CASP1, CASP9, ISGs (OAS2, MyD88, IFIT1, ISG15, TGFB1, IRF7, IL-8*, among others)	Antiviral response, Angiogenesis, Apoptosis	Histone deacetylation	Human fetal microglia, astrocytes	Suh et al. (2010)
STAT-1 dependent genes, ISGs	Antiviral response, apoptosis, anti-tumor response	Histone deacetylation	Colorectal carcinoma cells; L929 cells (mouse fibroblasts)	Génin et al. (2003), Klampfer et al. (2004)
*2′*–5′ OAS, ISG54, IFITM3, IP-10	Antiviral response	Histone deacetylation	2fTGH (sarcoma) cells	Nusinzon and Horvath (2003)
*CIITA*	Antigen presentation	Histone deacetylation	Mouse plasmacytoma cells; squamous cell carcinoma; rhabdomyosarcomas	Kanaseki et al. (2003), Chou (2005), Londhe et al. (2012)
unknown	Antigen presentation	Histone deacetylation	Mouse plasmacytomas	Khan et al. (2004)
*CIITA*	Antigen presentation	H3K27 trimethylation	Uveal melanoma cells, breast cancer cells	Holling et al. (2007), Truax et al. (2012)
*CIITA*	Antigen presentation	DNA hypermethylation	Head and neck cancer cells, choriocarcinoma cells, uveal melanoma, colorectal and gastric carcinomas	Morris et al. (2000), Satoh et al. (2004), Radosevich et al. (2007), Meissner et al. (2008)
*CIITA*	Antigen presentation	Histone deacetylation, DNA hypermethylation	Myeloid leukemia	Morimoto et al. (2004)
*TAP-1*	Antigen presentation	Histone H3 acetylation	Carcinomas	Setiadi et al. (2007)

*Numerous reports have cited instances of epigenetic modulation affecting permissibility to virus infection, many of which occur in tumor cells. Here we present a summary of these reports, listing the genetic target and its cellular function, the epigenetic modification, and the cell type involved. IFN, interferon; ISG, interferon stimulated gene; IFI, IFN alpha inducible protein; LMP, low molecular weight polypeptide; STAT, signal transducer and activator of transcription; CXCL1, C-X-C motif ligand 1; CREB3L1, cAMP responsive element binding protein 3 like-1; MX1, myxovirus resistance 1; IFIT1, interferon-induced protein with tetratricopeptide repeats 1; IRF, IFN regulatory factor; OAS, 2′–5′ oligoadenylate synthetase; TIP30, TAT-interacting protein 30; IL, interleukin; TRAIL, tumor necrosis factor- related apoptosis- inducing ligand; HLA, human leukocyte antigen; JAK1, janus kinase 1; DR4, Apo2/TRAIL receptor 4; XAF1, x-linked inhibitor of apoptosis-associated factor 1; ISRE, IFN sensitive response element; FGF2, fibroblast growth factor 2; VEGFC, vascular endothelial growth factor C; CASP, caspase; TGFB1, transforming growth factor beta 1; CIITA, Class II MHC transactivator; TAP-1, transporter 1, ATP-binding cassette, sub-family B.*

## THE ROLE OF EPIGENETICS IN HOST SUSCEPTIBILITY TO VIRAL INFECTION

Epigenetic regulation of innate and adaptive immune processes is emerging as a key determinant of susceptibility to viral infection. Several reports suggest that cell type-specific epigenetic regulation of antiviral ISGs leads to differences in permissibility to virus infections in both normal and tumor cells (Naka et al., 2006; Nguyen et al., 2008; Fang et al., 2012; Chen et al., 2013; Cho et al., 2013). Recently, histone H3K9 di-methylation, a repressive heterochromatin mark, was found to be elevated within IFN genes and ISGs in non-professional IFN-producing cells (e.g., fibroblasts) as compared to professional IFN-producing plasmacytoid dendritic cells (pDCs). Interestingly, inhibiting the KMT G9a by both genetic and pharmacological means led to increased IFN production and responsiveness in fibroblasts. In line with this, G9a-ablated fibroblasts were also rendered more resistant to infection by viruses (Fang et al., 2012; **Figure 1**).

Another recent study in mice harboring the murine viral susceptibility locus *Tmevp3 *revealed the intriguing role of *NeST*, a long non-coding RNA (lncRNA) adjacent to the IFN-γ locus in both mice and humans (Vigneau et al., 2001). NeST was found to function as an epigenetically driven enhancer element (Gomez et al., 2013) leading to increased IFN-γ production in mouse CD8^+^ T cells by directly interacting with the H3K4 histone methyltransferase complex and increasing H3K4 trimethylation, an activating mark. This novel epigenetic modification culminated in heightened susceptibility to persistent viral infection in mice (Gomez et al., 2013; **Figure 1**). Although the role of *NeST *in human epigenetic regulation is currently unknown, it is likely lncRNAs contribute to epigenetic regulation and manifestation of cell phenotypes including permissiveness to virus infection and cancer.

## CANCER EPIGENETICS IMPACT THE REGULATION OF ANTIVIRAL RESPONSE GENES

As previously discussed, the majority (but not all) of cancer cells are dysfunctional in their ability to produce and/or respond to IFN (Dunn et al., 2006). While crosstalk between oncogenic signals and the antiviral response pathways have been shown to play a role (Farassati et al., 2001; Shmulevitz et al., 2005); epigenetic events are also likely contributors to this phenotype. One indication of this comes from a series of studies on cells derived from cancer-prone Li-Fraumeni syndrome patients. Cells from these patients spontaneously immortalize when serially passaged in tissue culture due to mutations in the tumor suppressor p53, however transformation is inhibited upon treatment with 5-AZA (Kulaeva et al., 2003; Fridman et al., 2006). DNA methylation profiling of these immortalized cells revealed hypermethylation at the promoters of numerous genes involved in the type I IFN pathway, including IRF7 (Kulaeva et al., 2003; Fridman et al., 2006; Li et al., 2008). Interestingly, these immortalized Li-Fraumeni patient-derived cells were inherently more sensitive to VSV infection (Fridman et al., 2006; **Figure 1**).

Indeed, epigenetic repression of IFN and associated genes correlates with IFN insensitivity in many cancers. IRFs 4, 5, 7, and 8 are the target of DNA methylation, leading to dysfunctional responsiveness to type I and II IFNs in gastric cancer (Yamashita et al., 2010), while IRF8 is silenced by the same mechanism in several carcinomas (Lee et al., 2008). Similarly, IFN responsiveness was found to be suppressed in colon carcinoma cells due to DNA methylation at STAT1, STAT2, and STAT3, which can be restored following 5-AZA treatment (Karpf et al., 1999; **Figure 1**). Along the same signaling axis, epigenetic silencing of JAK1 in prostate adenocarcinoma cells was associated with unresponsiveness to both type I and type II IFNs (Dunn et al., 2005).

IFN-induced apoptosis is mediated by ISGs including Apo2L/TRAIL, which are also often dysfunctional in cancers (Reu et al., 2006b; Borden, 2007; Bae et al., 2008; Burton et al., 2013). Genes involved in Apo2L/TRAIL signaling, including TRAIL, the TRAIL receptor DR4, RASSF1A, and XAF1 are epigenetically silenced in melanomas (Reu et al., 2006a, b; Bae et al., 2008), leukemia (Soncini et al., 2013), renal carcinoma (Reu et al., 2006a) and experimentally transformed cells (Lund et al., 2011). Interestingly, 5-AZA treatment can restore TRAIL-mediated apoptosis induced by type I and II IFN (Reu et al., 2006a,b; Bae et al., 2008; Lund et al., 2011; Soncini et al., 2013; **Figure 1**). However, this cell death pathway is likely also epigenetically silenced through histone PTMs given that in medulloblastoma, IFN-γ could induce apoptosis via TRAIL only following treatment with the HDAC inhibitor valproic acid (Hacker et al., 2009).

Overall, these studies highlight multiple epigenetic mechanisms that transcriptionally repress IFN-associated genes, culminating in dysfunctional and non-responsive IFN signaling across various cancer subtypes. However, in some instances alterations to epigenetic modifications in cancer lead to the up-regulation of antiviral factors. In both gastric tumors and gliomas, overexpression of the ISG IFITM1 promotes cancer cell migration and invasion, and its elevated expression is linked to reduced CpG methylation levels (Yu et al., 2011; Lee et al., 2012). Alongside its oncogenic properties, IFITM1 has antiviral properties, through its ability to inhibit viral membrane fusion (Li et al., 2013; **Figure 1**).

It is also notable that while most cancers display IFN pathway defects, approximately a third of cancer cells are fully functional in their ability to produce and respond to IFN (Stojdl et al., 2003; Norman and Lee, 2000). Importantly, several studies have shown that HDAC inhibition using a variety of chemical inhibitors modulate IFN-induced expression of ISGs, type I IFN, and TLR3/4 (Génin et al., 2003; Nusinzon and Horvath, 2003; Chang et al., 2004; Klampfer et al., 2004; Sakamoto et al., 2004; Suh et al., 2010), which leads to increased OV activity in resistant cells (Nguyen et al., 2008). This further highlights the key role of epigenetic regulation in the generation of an antiviral response and suggests that it may be possible to improve OV efficacy in resistant tumors by manipulating the cancer epigenome as will be discussed shortly.

## CANCER CELLS EPIGENETICALLY REGULATE GENES INVOLVED IN ANTIGEN PRESENTATION

In addition to inactivating the antiviral response to escape anti-proliferative/pro-death signals, tumors must also evade immune recognition and clearance. To this end, many tumor types epigenetically suppress CIITA expression by mechanisms including histone deacetylation/methylation and DNA promoter methylation, resulting in suppressed IFN-γ mediated MHC-I and MHC-II gene expression and dysfunctional antigen presentation (Morris et al., 2000; Kanaseki et al., 2003; Morimoto et al., 2004; Satoh et al., 2004; Chou, 2005; Holling et al., 2007; Radosevich et al., 2007; Meissner et al., 2008; Londhe et al., 2012; Truax et al., 2012; **Figure 1**). Interestingly, treatment of cancer cells with HDAC inhibitors can promote antigen presentation and ultimately help to induce anti-tumor immunity (Khan et al., 2004; Chou, 2005). For example, trichostatin A (TSA)-treated irradiated B16 melanoma cells administered prophylactically as a cancer vaccine are significantly more effective then control irradiated B16 cells at protecting from a subsequent challenge with live B16 tumor cells (Khan et al., 2007). Cancer immune evasion can also be mediated by dampened expression of the transporter associated with antigen processing 1 (TAP1), a key factor for antigen presentation by MHC molecules (Johnsen et al., 1999). In carcinoma cells, decreased TAP1 expression was attributed to reduced levels of histone H3 acetylation at the TAP-1 promoter (Setiadi et al., 2007; **Figure 1**).

In addition to these direct epigenetic effects on components of the antigenic response within cancer cells, the tumor microenvironment has also been shown to epigenetically drive tumor infiltrating CD4^+^ T cells to tolerance. In colon cancer, infiltrating CD4^+^ lymphocytes displayed high levels of DNA methylation at the IFN-γ promoter, and consequently required treatment with 5-AZA to enable tumor antigen-stimulated IFN-γ production (Janson et al., 2008; **Figure 1**). Overall, these studies highlight the role of epigenetic control in conferring “stealth” status to tumor cells such that they may evade the immune surveillance.

## HDAC INHIBITORS CAN ALTER SUSCEPTIBILITY TO ONCOLYTIC VIRUSES

As alluded to earlier, defects in the IFN pathway are common in many malignancies but a significant proportion of tumors retain an active antiviral response (Stojdl et al., 2003; Dunn et al., 2006). Overcoming this antiviral response has been identified as a key barrier to the success of OV therapy and is the focus of many research groups including our own (Parato et al., 2005; Chiocca, 2008; Diallo et al., 2010; Liikanen et al., 2011; Russell et al., 2012). To overcome this barrier, many groups have looked at the possibility of using HDAC inhibitors in combination with OV therapy due to their repressive effects on the IFN-mediated antiviral response.

In one of the earliest reports, the anti-tumor effect of oncolytic adenovirus (OBP-301) in human lung cancer cells was found to synergize with FR901228 (Romidepsin), a class I HDAC inhibitor (Watanabe et al., 2006). However, in this report, increased activity was attributed to the upregulation of coxsackie adenovirus receptor (CAR) expression in cancer cells as opposed to direct effects on the antiviral response. Intriguingly, valproic acid, a class I/II HDAC inhibitor was found by another group in parallel to inhibit oncolytic adenovirus through the up-regulation of p21 (WAF1/CIP1; Hoti et al., 2006). Subsequently, TSA and valproic acid, two pan-HDAC inhibitors were found to enhance HSV oncolysis in squamous cell carcinoma and glioma cells (Otsuki et al., 2008; Katsura et al., 2009). Around the same time, Nguyen et al. (2008) showed that several HDIs could synergize with the oncolytic VSV-Δ51, an attenuated oncolytic VSV-mutant that is incapable of blocking IFN production (Stojdl et al., 2003). Combination treatment with HDIs resulted in synergistic cell killing, due to both enhanced induction of cell death and increased viral output (typically over 100-fold). Enhanced viral spreading of VV and semliki forest virus (SFV) was also observed in this study. Subsequent to this, TSA was shown to be particularly effective for improving VV-based OVs in several resistant cancer cell lines *in vitro* and in subcutaneous xenograft and syngeneic lung metastasis mouse models (MacTavish et al., 2011). Importantly, the impacts of HDAC inhibitors on OV spread and efficacy remain restricted to tumors and not normal cells, presumably because cancer cells exhibit a number of additional aberrations, such as increased metabolism, that promote viral growth independent of the status of the antiviral response.

## HDAC INHIBITORS AS MODULATORS OF ONCOLYTIC VIRUS-ASSOCIATED ANTI-TUMOR IMMUNITY

While initial experiences with HDAC inhibitors in combination with OVs exploited mainly the ability of these epigenetic modifiers to improve the infectivity of resistant tumors, at least in part by dampening the innate cellular antiviral response, more recent studies have further exploited the broader immunological effects of HDAC inhibitors. For example, one report showed that valproic acid suppresses NK cell activity by blocking STAT5/T-BET signaling leading to enhanced oncolytic HSV activity (Alvarez-Breckenridge et al., 2012). Also of note, a recent report by Bridle et al. (2013) demonstrated significant improvements in the generation of an anti-tumor immune response elicited against aggressive melanoma following a heterologous prime-boost vaccination strategy. After the establishment of intracranial melanomas, immune-competent mice were primed with a non-replicating adenovirus expressing the dopachrome tautomerase (hDCT) melanoma antigen, and then boosted with oncolytic VSV expressing hDCT. While this prolonged survival, mice were fully cured (64%) only when VSV-hDCT was administered in combination with the class I HDAC inhibitor MS-275. Remarkably, MS-275 reduced VSV-specific neutralizing antibodies and memory CD8^+^ T cells while maintaining prime-induced levels of humoral and cellular immunity against the tumor antigen. Interestingly, MS-275 also ablated autoimmune vitiligo typically observed following immunization against the melanocyte-expressed antigen (Bridle et al., 2013).

## USE OF OTHER EPIGENETIC MODULATORS TO IMPROVE ONCOLYTIC VIROTHERAPY?

Given the epigenetic regulation of the antiviral response and antigen presentation pathways, it is tempting to speculate that other epigenetic modulators, in addition to HDAC inhibitors, may also be used to amplify therapeutic responses in combination with OVs. To this end, a recent study by Okemoto et al. (2012) showed that**5-AZA treatment could enhance HSV replication when co-administered with IL-6 (**Figure 1**). However, given numerous reports of cancers epigenetically silencing antiviral genes by DNA methylation (**Table 1**), we would expect that in general 5-AZA and other DNA methyltransferase inhibitors should be ineffective at overcoming the cellular antiviral response. On the other hand, the advent of new pharmacological inhibitors of KMTs and KDMs brings forth new possibilities for improving OV efficacy. For example, given the finding that histone H3K9 dimethylation observed at ISGs correlates with repression and reduced IFN response/expression, investigating the potential utility of H3K9-demetylase inhibitors for enhancing OV spread in resistant tumors seems warranted. However, it is of critical importance that, as is observed for HDAC inhibitors, OV-enhancing effects remain tumor-selective.

## CONCLUSION

While genetic mutations are believed to be essential initiators of carcinogenesis, it is clear that epigenetic deregulation plays a key role in augmenting and/or maintaining the tumor phenotype. OVs are promising biotherapeutics that among others take advantage of the epigenetic silencing of cellular antiviral response genes and in many ways unmask cancer antigens as they destroy cancer cells and promote an inflammatory response. While additional studies on the impact of epigenetic regulation on the antiviral and immunological responses are needed, it is already recognized from studies using HDAC inhibitors that epigenetic modulators can positively impact OV efficacy. Additional *in vitro* and *in vivo* studies evaluating the effect of other epigenetic modulators are needed to determine whether these could be used in combination with promising OV platforms anticipated to reach the clinic in the near future, to further improve their therapeutic impact.

## Conflict of Interest Statement

John C. Bell is CSO and co-founder of Jennerex Biotherapeutics.
